# Ulna Autograft for Wrist Arthrodesis: A Novel Approach in Failed Wrist Arthoplasty

**DOI:** 10.2174/1874325001711010768

**Published:** 2017-08-21

**Authors:** Nastaran Sargazi, M. Philpott, A. Malik, M. Waseem

**Affiliations:** Department of Trauma and Orthopaedics, Macclesfield District General Hospital, Macclesfield, UK

**Keywords:** Failed wrist arthroplasty, Wrist arthrodesis, Ulna graft, Wrist fusion, Total wrist arthroplasty, Rheumatoid arthritis

## Abstract

Rheumatoid arthritis is a polyarthropathy affecting approximately 1% of the population worldwide. Wrist involvement is observed around 75% of patients, resulting in substantial disability and morbidity. A multidisciplinary approach to management of such patients is undertaken to prevent disease progression, many go on to develop debilitating disease requiring surgical intervention. Total wrist arthroplasty and arthrodesis are the main options available for those with end-stage disease, with arthroplasty preferred due to its ability to preserve a good degree of wrist function. Where complications occur with total wrist arthroplasty, salvage surgery with arthrodesis can be considered, however this requires satisfactory bone stock to enable stable fusion of the joint following arthroplasty. We report our experience of Ulna strut allografts in wrist arthrodesis in the management of failed total wrist arthroplasty.

## 1 INTRODUCTION

Rheumatoid arthritis (RA), a chronic systemic inflammatory autoimmune disorder of unknown aetiology, occurs in approximately 1% of the population worldwide [1] and commonly affects those aged 40-60 years, in particular females [2], who are regularly present with polyarthritis [1]. The disease process has a propensity to involve synovial joints [2], specifically the wrist, which affects 75% of patients, with 95% progressing to bilateral disease [1], thereby resulting in significant morbidity [1]. Deformities of the wrist is a major source of disability, requiring multiple surgical and reconstructive procedures which can be challenging, especially in those with limited tissue availability secondary to destructive, end-stage disease [3].

A limited number of procedures are available to address bony destruction, with total wrist arthroplasty (TWA) becoming an increasingly popular option due to its ability to address joint pain [4] whilst preserving movement and function [5]. TWA was first performed in 1890 by Themistocles Gluck using an ivory ball-in-socket designed implant, with modern designs now ranging from silicone to cobalt chrome and titanium implants [4]. Regardless of implant type, general principles involve fixation of the proximal component within the distal radius, with the distal component normally fixed into the distal carpal row and occasionally into the base of the metacarpals to provide additional stability [6]. Despite the drastic improvements in function, complications exist including loosening of the distal component, joint instability, dislocation, radioulnar impingement and carpal collapse in those with distal ulna resection [7].

Failed TWA poses a great challenge for upper limb surgeons, with the principle remaining option being arthrodesis of the wrist joint [8]. Such rigid fixation requires a good bone stock, which is normally not present in those with failed arthroplasty [9]. As a result, arthrodesis is traditionally accomplished by obtaining autogenous cancellous or corticocancellous bone grafts from the resected portions of the distal radius or most commonly, the iliac crest, thereby bridging the gap created between the distal radius and metacarpal bases following previous arthroplasty [9]. The morbidity associated with the harvest of bone graft from distant sites such as the iliac crest, and the lack of sufficient bone graft from the distal radius is a limiting factor in wrist arthrodesis, which has paved the way for new approaches to wrist fusion [9]. In this paper, we describe a new technique for wrist arthrodesis, using an ulna strut autograft, as a salvage procedure in those with failed arthroplasty for severe rheumatoid arthritis.

## 2 SURGICAL TECHNIQUE

An incision is made from the distal two thirds of the forearm, extending between the 3^rd^ and 4^th^ extensor compartment and ending at the distal portion of the 3^rd^ metacarpal. Skin, fat, fascia and extensor retinaculum are incised at the wrist, exposing the distal radius and ulna. Soft tissues and periosteum are mobilised from the radius proximally and from the dorsum of metacarpal distally, taking care to retract and preserve the overlying extensor tendons. The metacarpal screws and component are removed. The uncemented radial component of the arthroplasty is removed carefully using osteotomies.

The ulna is inspected. The ulna head and associated TFCC and surrounding soft tissues have previously been removed during the total wrist arthroplasty. The distal ulna diaphysis was osteomtomised approximately 5cm proximal to its distal extent. All soft tissues were mobilised from the graft apart from the pronator quadratus muscle to allow some vascularity to this portion of the bone. The blood supply to the pronator quadratus comes from the anterior interosseous artery, which runs down the forearm along the interosseous membrane [10]. The pronator quadratus was lifted from the distal radius but caution was used to ensure the pronator quadratus muscle was still attached to the interosseous membrane along with the anterior interosseous artery.

**Fig. (1):**
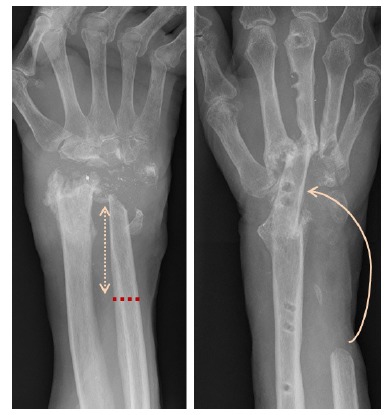
Surgical technique.

The proximal attachments act as pivot to allow 180 degree rotation of the strut graft between the radius and metacarpal (Fig. **1**). A standard fusion plate is fixed to the dorsum of the metacarpal, ulna graft and radial shaft using locking standard screws. A small volume of bone may also be used to provide additional packing at the proximal and distal ends of the strut graft. Repair of incised soft tissues are undertaken in layers.

## 3 CASE PRESENTATION

### a. Case 1

A 58-year old, right hand dominant factory worker with a background of previous left sided distal radial fracture and advanced Rheumatoid arthritis was referred to our local hand unit following failure of medical management of chronic wrist pain and progressive joint deformity. Clinically she was noted to have a subluxed left wrist with radiological features of advanced RA and malunion of the distal radial fracture. Furthermore, there was clinical evidence FDP rupture in the left middle and index fingers, in addition to extensive tenosynovitis of the middle finger and EPL rupture involving the right hand.

**Fig. (2):**
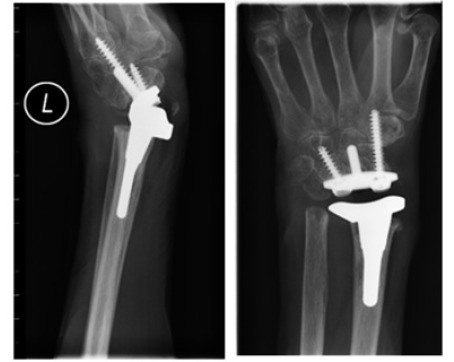
Post-operative imaging following left total wrist replacement.

Initial management involved addressing symptoms within her dominant hand with the patient undergoing dorsal wrist synovectomy and EIP tendon transfer to address the ruptured EPL tendon. Whilst she recovered well from the above procedures, symptoms within the left hand continued to deteriorate with the patient developing profound neuropathic pain in the left side. Clinically, she was now exhibiting evidence of muscle wasting within the left forearm alongside inability to flex all digits with ongoing deformity of the wrist. Therefore a left total wrist replacement was undertaken to address these issues (Fig. **2**).

**Fig. (3):**
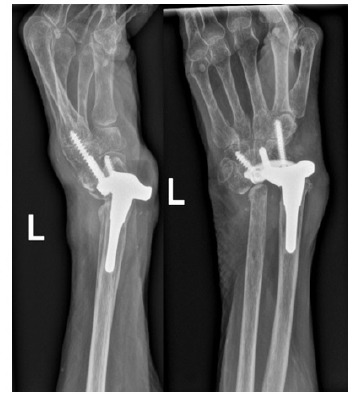
Dislocated total wrist replacement.

**Fig. (4):**
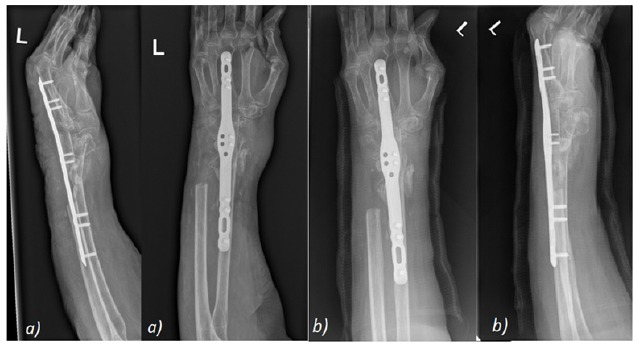
Wrist arthrodesis with ulna strut graft a) immediately post-op b) 2 months post-op.

Post-operatively, the patient exhibited a drastic improvement in both symptoms and function with ability to dorsiflex and plantarflex to 45^o^ and 20^o^ respectively, in addition to maintaining full supination and pronation. Unfortunately, she went on to develop atraumatic dislocation of the wrist replacement 8 years following the arthroplasty (Fig. **3**), henceforth underwent removal of the wrist replacement followed by arthrodesis of the wrist joint with an ulna strut graft (Fig. **4**).

**Fig. (5):**
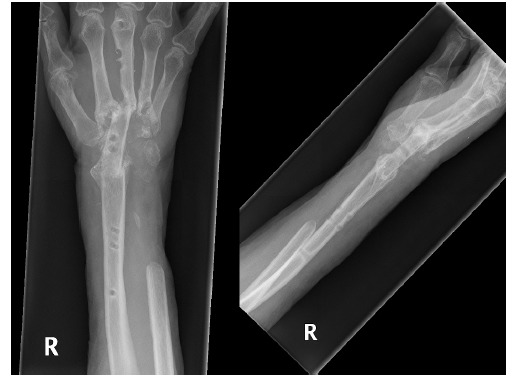
Removal of fusion plate at 13 months.

She progressed well post-operatively, with imaging at 2 months post-operatively illustrating evidence of graft take and bone healing. Approximately, 12 months following the arthrodesis, the patient's main complaint was prominence of the distal portion of the fusion plate and subsequent skin irritation. She subsequently underwent removal of the fusion plate at 13 month post-arthrodesis with intra-operative imaging and direct inspection of the fusion site showing evidence of complete bone healing and graft take (Fig. **5**). She continues to remain under follow-up.

### b. Case 2

**Fig. (6):**
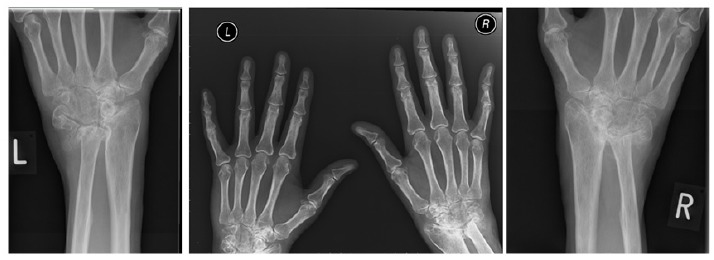
Pre-operative imaging showing bilateral wrist joint disease.

A 68-year old right hand dominant psychotherapist with a background of RA, numerous previous tenosynovectomy procedures to both hands and FPL tendon reconstruction was referred to our unit with progressive bilateral wrist pain and deformity. Clinical and radiological evidence of severe RA was noted bilaterally with z-deformity at the wrists and ulnar tranlocation of the carpus on the radio-ulnar joints (Fig. **6**).

**Fig. (7):**
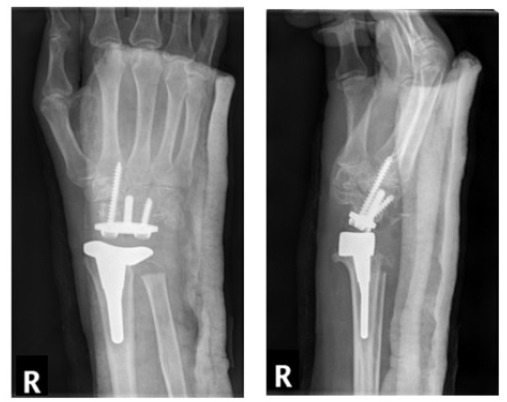
Post wrist arthroplasty imaging.

**Fig. (8):**
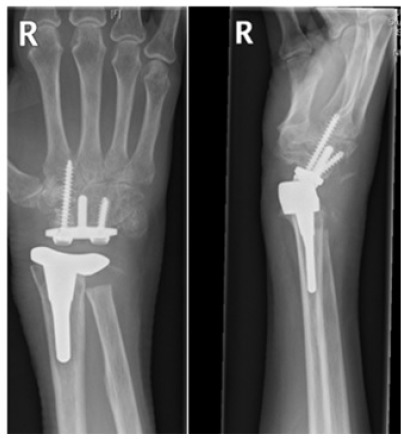
Wrist arthroplasty at 2 months.

**Fig. (9):**
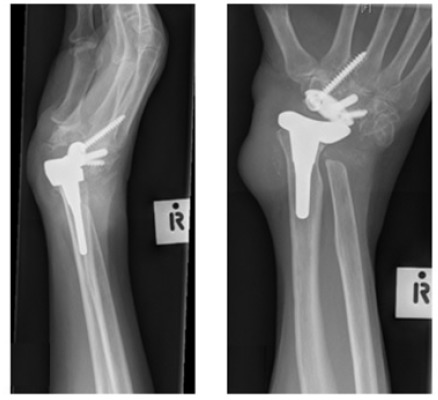
Progression of arthroplasty at 24 months.

**Fig. (10):**
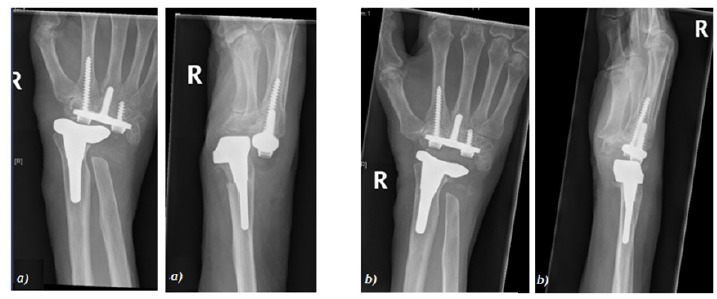
Dorsal dislocation of wrist a) Pre-MUA b) Post- MUA.

A right wrist arthroplasty was undertaken (Fig. **7**), with the patient demonstrating good post-operative function and ability to dorxsiflex and plantarflex to 50^o^ and 10^o^ at 2 months post-operatively (Fig. **8**). Unfortunatley, radiological evidence of dorsal progression of the distal prosthesis was evident at approximately 13 months post op, with the wrist replacement subluxing both clinically and radiologically at 24 months following the arthroplasty (Fig. **9**). Subsequently, the patient underwent revision of the right wrist arthroplasty, however presented with dorsal dislocation of the wrist 1 month later requiring manipulation under anaesthesia (MUA), with a good range of movement with improved grip in the hand post MUA (Fig. **10**).

**Fig. (11):**
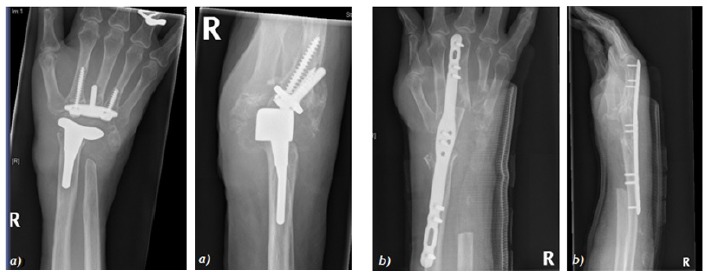
Conversion of wrist arthroplasty (a) to arthrodesis with ulna graft (b).

**Fig. (12):**
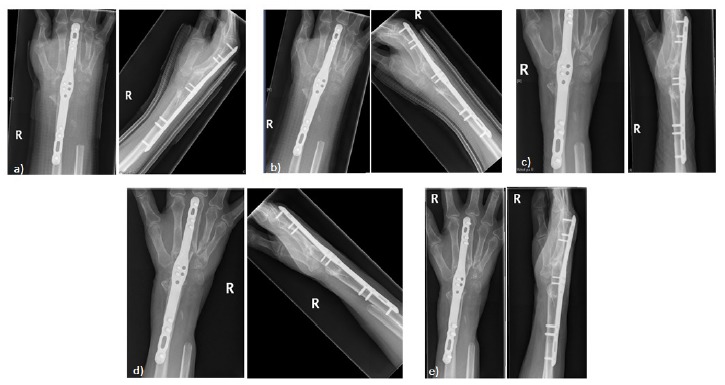
Post-operative imaging at a) 1 month b) 2 months c) 4 months d) 6 months e) 9 months following arthrodesis with ulna graft showing satisfactory fusion with bone graft take.

Despite the satisfactory radiological and clinical improvement, the patient went onto develop further migration of the distal component requiring a further revision. Unfortuantely, this did not resolve the issues relating to the migration of the prosthesis, thereby resulting in an eventual right wrist arthrodesis using an ulna bone graft in this patient (Fig. **11**). Follow-up imaging has shown satisfactory bone healing and integration of the ulna graft at the fusion site at 9 months post-op (Fig. **12**).

## DISCUSSION

Surgical intervention for RA in not merely dependent on radiological progression of the disease, but involes a spectrum of factors including patient age, functional demands, level of pain and response to previous soft tissue procedures [9]. Total wrist arthroplasty (TWA) has become a popular option in those with end-stage rheumatoid arthritis by allowing preservation of movement at the wrist joint, thereby providing patients with a degree of independence in relation to their activities of daily living [5].

Where TWA fails, revision radiocarpal arthroplasty is an option [8]. However, this is associated with a high failure rate and requires a more challenging and extensive reconstruction [8]. The remaining option in such patients is arthrodesis of the wrist joint, however this procedure is complicated by a limited availability of bone secondary to the destructive nature of the disease and the multiple previous reconstructive procedures [9]. The resultant outcome is a generlaised reduction in the overall range of movement in the joint, however, in those with debilitating diseaes, fusion of the wrist in a position of function may in fact improve function and motion of the digits [9].

Arthrodesis involves bridging of a fusion plate between the radius and its respective matacarpals, resulting in generalised reduction in the overall range of movement within the joint [9]. Whilst this is not ideal in healthy subjects, in those with debilitating disease, it enables fusion of the wrist in a position of function, which may in fact improve hand function and movement in the digits [9].

Unfortunately, such fusions are limited by the lack of a robust and sufficient bone stock, which is usually not present in this patient cohort due to reasons highlighted previously [9]. Therefore, autogenous cancellous or corticocancellous bone grafts from immediate and distant sources (*i.e.* distal radius or iliac crest respectively) are utilised to address such issue and enable rigid fixation of the plate [9]. Given the donor site morbidity associated with bone grafts from the iliac crest and the lack of sufficiently-sized and strong bone from the distal radius, other sites must also be considered [9].

In this paper, we have discussed the use of the ulna strut graft, harvested locally from the surgical site following removal of components of the TWA. Such technique can be utilised to provide a healthy, strong bone graft, of adequate size, to allow satisfactory fusion of the wrist without significant shortening and destabilisation of the wrist joint in those with failed wrist arthroplasties. As highlighted above, the ulna strut graft remained viable and intergrated well with surrounding bone to provide stable fusion at the wrist joint with minimal donor-site morbidities.

## CONCLUSION

Where failure of total wrist arthroplasty occurs in rheumatoid arthritis, arthrodesis of the wrist joint is the primary salvage procedure, which is limited by lack of bone stock. In this paper, we describe a new technique utilised to undertake wrist arthrodesis, which provides a solution for limitations related to tradition methods. This is the first paper that we are aware of which describes the use of such technique in wrist fusions. We therefore recommend this method as a salvage procedure in those with multiple failed arthroplasties and extensive joint disease resulting from end-stage RA.

## List Of ABBREVIATIONS

(EIP): = Extensor Indicis Proprius
(EPL): = Extensor Pollicis Longus
(FDP): = Flexor Digitorum Profundus
(FPL): = Flexor Pollicis Longus
(MUA): = Manipulation Under Anaesthesia
(RA): = Rheumatoid Arthritis
(TWA): = Total Wrist Arthroplasty
(TFCC): = Triangular Fibrocartilage Complex
